# Mechanisms of Mesenchymal Stem Cell‐Derived Exosomes in Dry Eye Disease: From Inflammation Pathways to Therapeutic Prospects

**DOI:** 10.1155/sci/2026673

**Published:** 2026-09-09

**Authors:** Jiahuan Yi, Fangfang Lu, Yale Tian, Yu Wang, Zihe Zhang, Xujie Deng, Zhi Wang, Shigang Xia, Jia Zhang, Qiguo Xiao

**Affiliations:** ^1^ Department of Ophthalmology, The Second Affiliated Hospital, Hengyang Medical School, University of South China, Hengyang 421001, Hunan, China, usc.edu.cn; ^2^ Department of Ophthalmology Cardiovascular Internal Medicine, Hunan Normal University Affiliated Hengyang City Central Hospital, Hengyang 421001, Hunan, China

**Keywords:** clinical translation, dry eye disease, inflammation, mesenchymal stem cell-derived exosome

## Abstract

Dry eye disease (DED) is a chronic ocular surface disorder triggered by tear film imbalance, in which inflammatory disruption of immune homeostasis constitutes the core pathological mechanism. Mesenchymal stem cell‐derived exosomes (MSC‐Exos) offer promising anti‐inflammatory therapeutic potential through immune modulation, tissue repair, and their inherently low immunogenicity. This review elucidates MSC‐Exos’ molecular regulation of DED inflammation, demonstrating their suppression of the TLR4/NF‐κB signaling axis, the IRAK1/TRAF6/NF‐κB cascade reaction, the STAT3 transcriptional regulatory network, and the NLRP3 inflammasome activation pathway, while revealing a synergistic mechanism through which MSC‐Exos ameliorate the ocular surface inflammatory microenvironment by modulating the Th17/Treg immune balance via the gut–eye axis and facilitating FBXW7‐mediated ubiquitination degradation pathways. Despite this therapeutic potential, clinical translation is hampered by exosomal heterogeneity, difficulties in standardization, and suboptimal delivery efficiency and long‐term efficacy. Future progress will require the integration of nanotechnology and gene editing to enhance therapeutic functionality, ultimately positioning MSC‐Exos as precision biologics for DED.

## 1. Introduction

Dry eye disease (DED) is a common, multifactorial chronic ocular surface disease caused by a tear film homeostasis imbalance [1, 2]. According to the epidemiological survey report of the Tear Film and Ocular Surface Society’s Dry Eye Workshop II [3], the global prevalence of DED ranges between 5% and 50%, showing significant geographical differences [4–6].A study conducted in 2024, which included 139,556 samples [7], demonstrated that the overall prevalence of DED in Asian regions reached 23.9%, with an age‐related increasing trend, making DED one of the leading ocular diseases affecting quality of life. Persistent tear film instability, ocular surface inflammation, and sensory nerve abnormalities are hallmarks of DED [8]. Among these, abnormal expression of ocular surface inflammatory mediators and immune homeostasis imbalance play critical roles in the occurrence and progression of DED, forming the core mechanism of its vicious cycle [9, 10]. Accordingly, anti‐inflammatory intervention has become a cornerstone of DED treatment, and the development of safe and effective anti‐inflammatory agents remains a major research focus in ophthalmology.

In recent years, mesenchymal stem cell‐derived exosomes (MSC‐Exos) have attracted considerable attention in the treatment of inflammatory diseases owing to their roles in immune regulation, tissue repair, and intercellular signal transduction [11]. MSC‐Exos can regulate the immune microenvironment through various signaling pathways, suppress ocular surface inflammatory responses, and promote restoration of tear film homeostasis, thereby ameliorating the pathological state of DED [12]. Furthermore, MSC‐Exos have a high cargo‐loading capacity and are readily modifiable, offering broad prospects for the treatment and diagnosis of DED [13]. This review focuses on recent advances in MSC‐Exos‐mediated suppression of DED inflammation, systematically summarizing the roles of key signaling pathways and targets—including TLR4, STAT3, and NLRP3—and evaluating the therapeutic potential and current challenges of MSC‐Exos in clinical DED management, with the aim of providing theoretical support and research direction for precision treatment of DED. The detailed literature search strategy and study selection criteria are provided in Appendix S1.

## 2. DED and Ocular Surface Inflammation

DED is a highly heterogeneous chronic ocular surface disease. Patients commonly present with symptoms including dryness, redness, foreign body sensation, pain, photophobia, increased secretions, itching, and ocular fatigue. Ocular surface inflammation is the core driving factor in the pathogenesis of DED. The inflammatory response in DED mainly includes innate immune responses, adaptive immune responses, and neurogenic inflammation [14]. A range of internal and external factors—including hyperosmolarity, local infection, immune dysregulation, and hormonal changes—can disrupt the ocular surface microenvironment and trigger immune activation [15].

During the acute phase of DED, the innate immune system mounts the primary response to changes in the ocular surface microenvironment. Corneal epithelial cells serve as the first line of defense and rapidly respond to external stimuli by secreting matrix metalloproteinases (MMPs), chemokines, tumor necrosis factor‐α (TNF‐α), and interleukins (ILs). These cytokines and MMPs recruit and activate ocular surface neutrophils and natural killer (NK) cells, which attack damaged cells and amplify the inflammatory response [16]. Additionally, NK cells also secrete interferon‐γ (IFN‐γ) and other pro‐inflammatory factors that promote the maturation of antigen‐presenting cells (APCs) [17]. In DED, the principal APCs involved in innate immunity—dendritic cells, monocytes, and macrophages—subsequently drive adaptive immune responses [18] and gradually become the dominant response in the chronic stage of DED. Mature APCs present antigens to T cells, inducing differentiation into Th17 cells (which secrete IL‐17) and Th1 cells (which secrete IFN‐γ). These effector T cells migrate to the ocular surface, releasing cytokines that sustain inflammation and damage the epithelium [19]. Activated T cells also engage B cells through direct contact and cytokine signaling, promoting B cell proliferation and differentiation into plasma cells that secrete autoantibodies—further destabilizing the ocular surface microenvironment [20, 21].

The pro‐inflammatory microenvironment established by infiltrating immune cells damages not only the epithelium but also the normal structure and function of corneal nerves [22]. Under inflammatory stimulation, corneal epithelial cells produce and secrete neurotrophic factors that drive aberrant corneal nerve growth, alter subbasal nerve density, and can ultimately lead to nerve degeneration [23], manifesting as sensory abnormalities in DED patients. Simultaneously, corneal nerves are persistently activated in response to elevated osmotic pressure and epithelial injury, promoting neuropeptide release from nerve endings, increasing local inflammatory mediators, and thereby establishing neurogenic inflammation—a key component of the DED vicious cycle [24].

**Figure 1:**
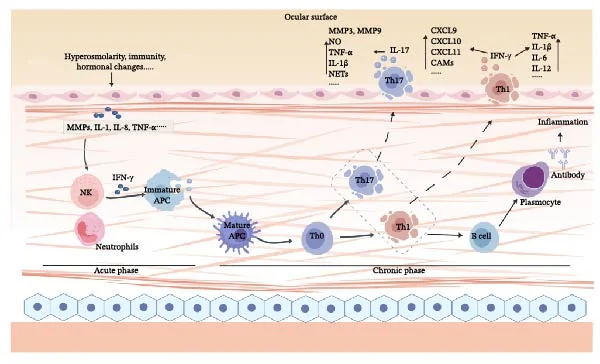
Acute‐to‐chronic inflammatory cascade on the ocular surface in dry eye disease. Tear hyperosmolarity, immune dysregulation, hormonal alterations, and other stressors activate ocular surface epithelial cells, inducing the release of matrix metalloproteinases and proinflammatory cytokines. During the acute phase, these mediators recruit and activate innate immune cells, including natural killer cells and neutrophils, and promote antigen‐presenting cell maturation. During the chronic phase, mature antigen‐presenting cells drive naïve CD4^+^ T cells toward T helper 1 and T helper 17 phenotypes. Activated T cells migrate to the ocular surface and release interferon‐γ and interleukin‐17, thereby increasing the expression of chemokines, cell adhesion molecules, matrix metalloproteinases, and additional inflammatory mediators. B cell activation and differentiation into plasma cells further promote antibody‐mediated inflammation, collectively sustaining the inflammatory cycle of dry eye disease. Arrows indicate cellular activation, differentiation, migration, or mediator release. APC, antigen‐presenting cell; CXCL, C‐X‐C motif chemokine ligand; DED, dry eye disease; IFN‐γ, interferon‐γ; IL, interleukin; MMP, matrix metalloproteinase; NETs, neutrophil extracellular traps; NK, natural killer; NO, nitric oxide; Th0, naïve CD4^+^ T cell; Th1, T helper 1 cell; Th17, T helper 17 cell; TNF‐α, tumor necrosis factor‐α.

In summary, the inflammatory mechanism of DED is complex (Figure 1). The ocular surface inflammatory microenvironment does not arise from a single mechanism but instead reflects the coexistence of multiple inflammatory processes at different stages of DED pathology, responding to evolving changes in the ocular surface microenvironment.

## 3. Role of MSC‐Exos in Suppressing Dry Eye Inflammation

### 3.1. Function of MSC‐Exos

MSC‐Exos are nanovesicles actively secreted by mesenchymal stem cells, ~30–150 nm in diameter, with a typical phospholipid bilayer structure and carrying various bioactive molecules, including proteins, lipids, mRNA, miRNA, and long noncoding RNA [25]. These exosomes play crucial roles in intercellular communication, tissue repair, and immune regulation. Through their nucleic acid and protein cargo, MSC‐Exos modulate gene expression and signal transduction in recipient cells, thereby regulating key biological processes such as cell proliferation, differentiation, apoptosis, and immune and inflammatory responses [26]. MSC‐Exos interact with recipient cells via membrane fusion, receptor‐mediated endocytosis, or direct content release following phagocytosis [27]. MSC‐Exos affect the functions of immune cells, including macrophages, T lymphocytes, B lymphocytes, dendritic cells, and NK cells, regulating the release of inflammatory factors [28–30]. Compared with MSCs themselves, MSC‐Exos exhibit lower immunogenicity, greater biostability, and easier storage, conferring substantial advantages in the treatment of inflammatory diseases, tissue repair, and immune modulation [31–35].

### 3.2. MSC‐Exos Exert Anti‐Inflammatory Effects by Targeting Immune Cells in the DED

Multiple studies have now established that MSC‐Exos regulate both T cell‐ and innate immune cell‐driven inflammatory responses in DED. For example, in DED mice, MSC‐Exos increase the production of immunosuppressive IL‐10, inhibit the differentiation of ocular surface neutrophils and monocytes into inflammatory phenotypes, promote the polarization of macrophages towards the M2 anti‐inflammatory type, antagonize T cell‐driven inflammatory responses, and establish an ocular surface immunosuppressive environment [36]; MSC‐Exos can also inhibit the differentiation of T cells into Th17, increase the expansion of regulatory T cells (Tregs) with immunosuppressive properties, alleviating inflammation mediated by Th17 cells [37]. Although the precise mechanisms by which B cells contribute to DED progression remain incompletely understood, preliminary evidence suggests that MSC‐Exos may suppress B cell‐mediated inflammatory responses driven by autoantibody secretion. PRDM1 (PR domain zinc finger protein 1) is a key regulator of B cell recruitment and inflammatory cytokine production and serves as a key regulator of B cell differentiation into antibody‐producing plasma cells. MSC‐Exos can promote the degradation of PRDM1, regulating B cell recruitment and differentiation, thereby weakening B cell‐related immune responses [21]. Elucidating the molecular signaling mechanisms underlying these cellular effects will facilitate the development of more effective, selective, and safe MSC‐Exos‐based therapeutics for DED.

## 4. Signal Mechanisms of MSC‐Exos in Suppressing Dry Eye Inflammation

### 4.1. TLR4/NF‐κB

Toll‐like receptors (TLRs) are widely present in mammalian cells, acting as pattern recognition receptors (PRRs) in the innate immune system that identify pathogen‐associated molecular patterns (PAMPs) or damage‐associated molecular patterns (DAMPs), inducing innate immune responses and triggering the release of inflammatory mediators and cytokines [38, 39]. Depending on the specific recognition of adapter proteins, TLR signaling pathways can be divided into myeloid differentiation factor 88 (MyD88)‐dependent or MyD88‐independent pathways, with most TLRs utilizing the MyD88‐dependent pathway [40]. Nearly all TLRs are expressed in the cornea and conjunctiva, and multiple TLR subtypes have been linked to DED pathology [41]. Among them, TLR4 has been studied most extensively in DED research, with studies proving that dynamic changes in TLR4 subcellular localization are key aspects of the DED pathological mechanism. Under normal physiological conditions, TLR4 is confined to the cytoplasm of corneal epithelial cells, whereas in DED, TLR4 translocates to the cell membrane without a corresponding increase in cytoplasmic TLR4 mRNA. This redistribution suggests that DED induces membrane translocation of TLR4 in mouse corneal epithelial cells [42], efficiently binding with DAMPs, triggering the “trigger” of ocular surface inflammation, and initiating downstream inflammatory signal cascades.

TLR4 also exacerbates ocular surface inflammation by impairing autophagy in corneal epithelial cells. In DED patients, the expression levels of pro‐inflammatory factors (calcium‐binding protein S100A9) secreted by neutrophils and macrophages increase [43, 44]. A new study found that besides directly promoting inflammatory responses, S100A9 can also activate TLR4, interfering with the normal autophagy of mouse corneal epithelial cells, exacerbating ocular surface inflammatory responses, and promoting corneal epithelial cell death [45].

The central role of TLR4 in DED inflammation has inspired several targeted therapeutic strategies. For instance, cyclosporine A targets TLR4 to reduce the transcription levels of downstream factors such as NF‐κB [46]; acteoside regulates the TLR4/PI3K axis to promote the production of immunosuppressive IL‐10 by human B cells [47], respectively, enhancing immune tolerance and suppressing inflammatory outbreaks. MSC‐Exos have similarly been shown to attenuate DED inflammation through the TLR4/innate immune axis. Bone marrow‐derived MSC‐Exos (BM‐MSC‐Exos) deliver miR‐21‐5p to ocular surface tissues, where it targets and suppresses TLR4 expression, thereby blocking the MyD88/NF‐κB signaling cascade and reducing pro‐inflammatory cytokine production. Importantly, this intervention not only resolves inflammation but also restores neuro‐immune homeostasis, increases tear secretion, and promotes conjunctival goblet cell proliferation to repair the ocular surface barrier—achieving multidimensional therapeutic effects in DED [37].

### 4.2. IRAK1/TRAF6/NF‐κB

The IRAK1/TRAF6/NF‐κB signaling pathway serves as the core regulatory hub for innate immunity and inflammatory responses [48, 49]. Its cascade activation plays a crucial role in the pathological process of DED. Upon ocular surface exposure to stimuli such as hyperosmolarity or oxidative stress, TLRs—including TLR4—are activated and recruit the adaptor protein MyD88, which subsequently triggers the phosphorylation of downstream IL‐1 receptor‐associated kinase 1 (IRAK1).

The activated IRAK1 binds to TNF receptor‐associated factor 6 (TRAF6). After self‐ubiquitination, TRAF6 activates a complex containing TGF‐beta activated kinase 1 binding protein 2 (TAB2) and induces the phosphorylation of IκB kinase, leading to the activation of NF‐κB. This transcription factor then translocates into the nucleus and initiates the gene expression of pro‐inflammatory cytokines such as IL‐6 and TNF‐α [40, 50].

In response to this inflammatory regulatory mechanism, MSC‐Exos exhibit unique therapeutic potential. Studies have shown that in a DED mouse model, MSC‐Exos can precisely intervene in the IRAK1/TAB2/NF‐κB pathway by delivering various microRNAs, such as miR‐125b‐5p and miR‐199‐3p. These microRNAs target and bind to the mRNAs of IRAK1 and TAB2, inhibiting their protein translation and thereby blocking the activation of NF‐κB and the release of downstream pro‐inflammatory cytokines [51]. Among the miRNAs carried by MSC‐Exos, miR‐146a has been the most extensively investigated in the context of innate immunity and inflammatory regulation. For example, MSC‐Exos can suppress the development of allergic airway inflammation by delivering miR‐146a [52]; human umbilical cord MSC‐Exos (UC‐MSC‐Exos) regulate microglial pyroptosis and autophagy through the action of miR‐146a, thus alleviating inflammatory pain [53].

To identify the active components in MSC‐Exos responsible for DED treatment, comparative analyses of blood samples from DED patients and healthy controls have demonstrated that IRAK1 is a direct target of miR‐146a, and that miR‐146a suppresses IRAK1 expression to attenuate DED‐associated inflammation [54]; Han et al. [55] applied miR‐146a mimics and inhibitors in human corneal epithelial cells and found that as the expression of miR‐146a increased or decreased, the IRAK1/TRAF6/NF‐κB pathway and downstream inflammatory factors showed an inverse trend. Therefore, miR‐146a can negatively regulate DED inflammation through the IRAK1/TRAF6/NF‐κB signaling pathway [55]. Collectively, these findings establish the mechanistic basis for developing MSC‐Exos‐based nanomedicines targeting this pathway and underscore the therapeutic potential of epigenetic regulation in ocular surface disease.

### 4.3. STAT3

The Janus kinase/signal transducer and activator of transcription (JAK/STAT) pathway is a ubiquitous intracellular signaling cascade that transmits extracellular signals through three essential components: tyrosine kinase‐associated receptors, JAK proteins, and STAT proteins [56, 57]. STAT3, a critical member of this pathway, is actively involved in inflammatory and immune responses across various pathological processes [58–60]. In DED, STAT3 regulation shows pronounced disease subtype‐specific differences. In Sjögren’s syndrome (SS)‐associated DED, studies have found that the calcium‐binding proteins S100A8 and S100A9, which are positively correlated with the severity of DED, promote dendritic cell‐driven Th17 cell immune responses by activating the STAT3 pathway, thereby exacerbating autoimmune inflammation at the ocular surface [61]. Conversely, in non‐SS DED, STAT3 demonstrates a dual regulatory role. Studies reveal that inhibition of STAT3 activation can mitigate corneal neovascularization, corneal opacity, and conjunctival inflammation caused by chronic inflammatory infiltration [62] yet paradoxically accelerates corneal epithelial cell apoptosis and exacerbates ocular surface damage [63]. This bidirectional functionality underscores the spatiotemporally specific regulatory role of STAT3 across different pathological stages of DED.

Despite the complexity of STAT3’s regulatory mechanisms in DED, MSC‐Exos show promising therapeutic potential through this pathway. For instance, in chronic graft‐versus‐host disease (GVHD)‐associated dry eye—a condition characterized by severe ocular pain, vision impairment, and refractory inflammation—topical application of MSC‐Exo eye drops improves tear film stability and increases tear secretion volume. Mechanistic studies further demonstrate that miR‐204 within MSC‐Exos not only binds to the 3^′^ untranslated region of the IL‐6 receptor gene to inhibit its translation but also directly suppresses IL‐6 production and STAT3 activation. This dual action drives the phenotypic transition of pro‐inflammatory M1 macrophages to anti‐inflammatory M2 macrophages, ultimately reducing corneal inflammatory factor release and promoting tissue repair [64].

### 4.4. NLRP3 Inflammasome

The innate immune system serves as the primary defense barrier against pathogenic invasion, with its activation contingent upon germline‐encoded PRRs. These receptors initiate rapid immune responses by detecting PAMPs or DAMPs, encompassing invading microorganisms, necrotic cells, or environmental toxicants, thereby maintaining tissue homeostasis [65]. Within this framework, the NOD‐like receptor family pyrin domain‐containing 3 (NLRP3) inflammasome functions as a pivotal effector complex in innate immunity. It orchestrates microbial clearance and tissue repair processes through the activation of downstream inflammatory cascades [66, 67].

The pathological manifestations of DED are frequently accompanied by hyperosmolar stress at the ocular surface. This microenvironment markedly enhances the assembly and activation of NLRP3 inflammasomes in conjunctival tissues, driving aberrant release of local pro‐inflammatory cytokines (e.g., IL‐1β and IL‐18), which subsequently exacerbate corneal epithelial barrier dysfunction and neurogenic inflammation [68]. Notably, dysregulation of NLRP3 inflammasome activity not only amplifies inflammatory progression in DED but also directly induces ocular surface tissue damage [69]. Investigations by Wang et al. [36] demonstrated significant upregulation of NLRP3 inflammasome components and downstream effectors (caspase‐1, IL‐1β, and IL‐18) at both transcriptional and protein levels in DED patients and animal models. Genetic silencing or pharmacological inhibition of NLRP3 effectively attenuated ocular surface inflammation and improved tear film stability [36].

Emerging evidence highlights the therapeutic potential of adipose‐derived MSC‐Exos (AD‐MSC‐Exos) in precisely modulating innate immune responses. Experimental studies revealed that topical administration of MSC‐Exos in DED murine models significantly suppressed NLRP3 inflammasome activation through epigenetic regulation mechanisms (e.g., miRNA delivery), concomitant with downregulation of caspase‐1, IL‐1β, and IL‐18 expression at the mRNA and protein levels [36]. This therapeutic effect may originate from exosomal cargo‐mediated interference with critical nodes in NLRP3 assembly, including apoptosis‐associated speck‐like protein containing a CARD (ASC) oligomerization and pro‐caspase‐1 cleavage. Such interventions effectively block IL‐1β/IL‐18 maturation and secretion, ultimately achieving the dual therapeutic benefits of inflammation resolution and tissue regeneration.

Candidate cargo molecules include miR‐223‐3p, which directly targets the 3^′^ untranslated region of NLRP3 mRNA and is markedly reduced in DED cornea/conjunctiva and patient samples [68] and MSC‐sourced miR‐146a, which has been implicated in restraining paracrine NLRP3 activation in inflammatory eye disease [70]. However, direct evidence linking these microRNAs to MSC‐Exos‐mediated NLRP3 suppression specifically in DED remains to be established.

### 4.5. Gut–Eye Axis

Gut microbiota homeostasis is a cornerstone of the host immune equilibrium and systemic health. Emerging evidence reveals that gut dysbiosis synergistically drives ocular surface disease progression through the gut–eye axis mechanism in conjunction with systemic inflammatory disorders [71, 72]. In SS patients, DED severity correlates inversely with gut microbiota α‐diversity, characterized by an elevated abundance of pro‐inflammatory microbial taxa and diminished anti‐inflammatory species [73–75]. Animal studies further demonstrate that exogenous supplementation of anti‐inflammatory probiotics (*Lactobacillus acidophilus* and Lactobacillus propionici) significantly alleviates ocular symptoms in SS murine models [76]. This immunomodulatory effect operates through dual mechanisms: vagus nerve‐mediated neurostimulation by gut microbial metabolites and metabolite‐driven modulation of Th17/Treg balance, collectively regulating systemic immune responses [77].

Current clinical management of SS predominantly relies on symptomatic interventions, lacking disease‐modifying therapies. Although MSCs have shown efficacy in attenuating autoimmune responses and inflammatory cell infiltration in SS, intravenous administration carries risks such as pulmonary capillary occlusion due to the large cell size [78]. In contrast, MSC‐Exos circumvent these limitations and safely ameliorate SS phenotypes by rebalancing Th17/Treg ratios and restoring gut microbiota homeostasis via the gut–eye axis. A pivotal study by Zou et al. [79] revealed that in spontaneous SS model mice, systemic delivery of UC‐MSC‐EXOs combined with splenic T cells increased salivary flow, elevated splenic Treg proportions, reduced Th17 populations, and enhanced gut microbial richness and evenness. In this study, systemic MSC‐Exos administration improved salivary gland function by rebalancing two distinct T cell populations and remodeling the gut microbiota in mice.

Notably, the target specificity of MSC‐Exos in orchestrating these effects remains elusive. Given the shared pathophysiological features between salivary and lacrimal glands in SS—including AQP5‐dependent secretory dysfunction and lymphocytic infiltration—it is hypothesized that MSC‐Exos may exert analogous therapeutic effects on lacrimal gland function through identical molecular pathways [80, 81]. However, this hypothesis requires further experimental validation, including tear osmolarity measurement and conjunctival goblet cell density analysis.

### 4.6. FBXW7

As the core substrate recognition subunit of the E3 ubiquitin ligase complex, F‐box and WD repeat domain‐containing 7 (FBXW7) exerts critical functions in cell cycle checkpoints, genome stability maintenance, and tumor suppression by mediating ubiquitination and degradation of key regulatory proteins [82, 83]. Emerging evidence highlights its regulatory role in inflammatory responses. For instance, Wu et al. [84] demonstrated that in a murine model of allergic rhinitis, overexpression of FBXW7 could reverse the cellular inflammatory response induced by the upregulation of miR‐223‐3p. Conversely, FBXW7 overexpression alleviates endothelial dysfunction and associated inflammation during atherosclerosis progression in mice [85]. Wang’s team recently extended this anti‐inflammatory mechanism to DED. Their study revealed that AD‐MSC‐Exos significantly ameliorate ocular surface damage, enhance goblet cell density, and reduce pro‐inflammatory cytokines in DED mice. Mechanistically, miR‐223‐3p enriched in AD‐MSC‐Exos binds to the 3^′^ untranslated region of FBXW7 mRNA, thereby suppressing FBXW7 expression and attenuating ocular surface inflammation [86]. This work identifies FBXW7 for the first time as a pivotal regulatory node in the inflammation‐repair imbalance of DED. However, the downstream signaling alterations resulting from miR‐223‐3p/FBXW7 axis activation remain to be elucidated, and further investigation is needed to clarify the precise anti‐inflammatory mechanisms of this axis.

### 4.7. Cross‐Regulatory Network of Pathways

**Figure 2:**
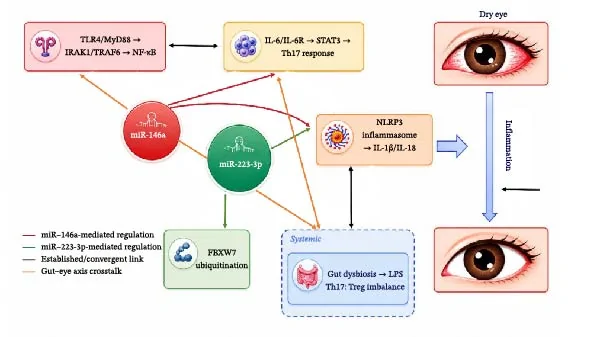
Cross‐regulatory network of MSC‐Exos‐modulated pathways in dry eye disease. miR‐146a and miR‐223‐3p represent putative shared nodes linking local innate inflammatory pathways with systemic gut–eye axis signaling. Red lines indicate miR‐146a‐mediated regulation; green lines, miR‐223‐3p‐mediated regulation; black lines, established or convergent links; and orange lines, gut–eye axis crosstalk. DED, dry eye disease; FBXW7, F‐box and WD repeat domain‐containing 7; IL, interleukin; IL‐6R, interleukin‐6 receptor; IRAK1, interleukin‐1 receptor‐associated kinase 1; JAK, Janus kinase; LPS, lipopolysaccharide; miR, microRNA; MSC‐Exos, mesenchymal stem cell‐derived exosomes; MyD88, myeloid differentiation primary response 88; NF‐kB, nuclear factor kB; NLRP3, NOD‐like receptor family pyrin domain‐containing 3; STAT3, signal transducer and activator of transcription 3; Th17, T helper 17 cell; TLR4, Toll‐like receptor 4; TRAF6, tumor necrosis factor receptor‐associated factor 6; Treg, regulatory T cell.

The six pathways summarized above (Sections 4.1–4.6) do not act as isolated modules; several core molecules operate at more than one node simultaneously, and the local innate‐immune cascades intersect with the systemic gut–eye axis at defined points. Two MSC‐Exos‐derived microRNAs, miR‐146a and miR‐223‐3p, occupy the most central positions in this network (Figure 2).

miR‐146a illustrates this cross‐pathway synergy most clearly. By directly targeting IRAK1 and TRAF6, MSC‐Exos‐delivered miR‐146a interrupts the TLR4/MyD88 cascade and blunts NF‐κB activation [54, 55]. Because NF‐κB‐driven IL‐6 also feeds the IL‐6R/JAK/STAT3 axis—a reciprocal relationship described as the “IL‐6 amplifier,” in which NF‐κB and STAT3 activation mutually reinforce one another [87]—suppression of IRAK1/TRAF6 by miR‐146a is predicted to secondarily dampen STAT3 activation. This hypothetical indirect effect may complement the direct miR‐204‐mediated inhibition of IL‐6R/STAT3 described in Section 4.3 [64]. miR‐146a has additionally been shown, in other inflammatory eye diseases, to directly target NLRP3 and ASC [70], suggesting that it may restrain the TLR4/NF‐κB and NLRP3 arms and, hypothetically, the STAT3 arm. Its IRAK1/TRAF6‐ and NLRP3‐suppressive actions are supported by direct evidence in ocular models [54, 55, 70], whereas the predicted effect on STAT3 is inferred from the shared NF‐κB/IL‐6 node, has not been measured directly in DED, and requires experimental confirmation.

miR‐223‐3p constitutes a second bridging node: the same microRNA that targets NLRP3 to limit inflammasome assembly (Section 4.4) [68] also targets FBXW7 to modulate the ubiquitination‐degradation pathway (Section 4.6) [86], thereby linking inflammasome control to protein‐stability regulation within a single cargo molecule. Notably, FBXW7 has demonstrated anti‐inflammatory activity in other experimental settings by restraining NF‐κB/IRF3 nuclear translocation [88], whereas suppression of FBXW7 by exosomal miR‐223‐3p was associated with reduced ocular surface inflammation in DED [86]. This apparent paradox may reflect tissue‐ and context‐dependent regulation because the net inflammatory effect of FBXW7 can vary according to cell type, inflammatory stimulus, disease stage, and the dominant ubiquitination substrates. Ocular surface‐specific downstream targets, together with parallel targets of miR‐223‐3p, may therefore contribute to the anti‐inflammatory phenotype observed in DED; however, this interpretation remains to be experimentally tested.

The gut–eye axis (Section 4.5) is not confined to a separate systemic compartment; it converges with the local TLR4/NLRP3 cascade at several points. Gut dysbiosis raises circulating lipopolysaccharide, which is recognized by TLR4 on ocular surface cells, providing a systemic input into the same cascade that MSC‐Exos‐derived miR‐21‐5p and miR‐146a target locally [77]. Conversely, the Th17/Treg imbalance corrected via the gut–eye axis [79] overlaps mechanistically with the local Th17‐skewing effects of STAT3 activation [61], suggesting systemic and local Th17 pools are reinforced by shared upstream signals rather than regulated independently. A further, currently hypothetical, link is that NLRP3‐derived IL‐1β/IL‐18 may reinforce Th17 differentiation and feed back into this systemic balance—documented in other autoimmune settings but not yet tested directly in DED.

Taken together, these convergences indicate that MSC‐Exos do not regulate TLR4/NF‐κB, IRAK1/TRAF6, STAT3, NLRP3, FBXW7‐ubiquitination, and the gut–eye axis as six parallel, independent pathways. Instead, miR‐146a and miR‐223‐3p act as shared regulatory nodes coupling innate and adaptive arms of local inflammation, while the gut–eye axis supplies systemic reinforcement of these same nodes—reframing the therapeutic logic of MSC‐Exos from independent multitarget action to convergent multinode regulation (Figure 2).

## 5. Opportunities and Challenges

### 5.1. Mechanistic and Methodological Limitations

**Figure 3:**
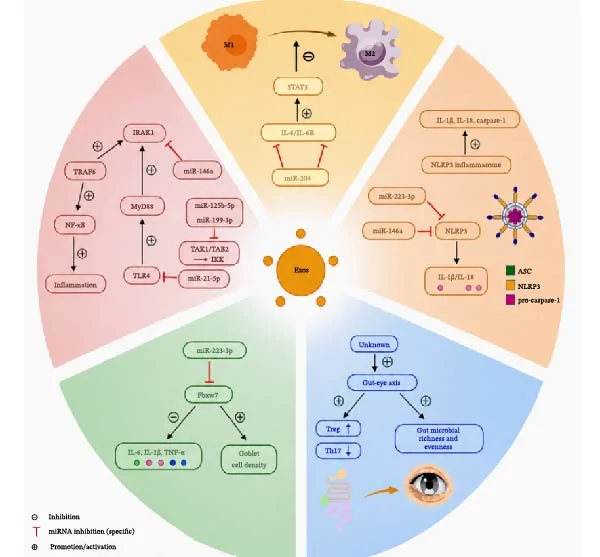
The signaling mechanism of MSC‐Exos in inhibiting DED inflammation. MSC‐Exos modulate the TLR4/MyD88/IRAK1/TRAF6/NF‐κB and IRAK1/TAB2/IKK pathways, the IL‐6/IL‐6R/STAT3 axis, NLRP3 inflammasome activation, the miR‐223‐3p/FBXW7 axis, and gut–eye axis‐associated Th17/Treg balance. Red blunt‐ended lines indicate specific microRNA‐mediated inhibition; plus and minus signs indicate activation and inhibition, respectively. ASC, apoptosis‐associated speck‐like protein containing a caspase recruitment domain; DED, dry eye disease; FBXW7, F‐box and WD repeat domain‐containing 7; IKK, inhibitor of nuclear factor κB kinase; IL, interleukin; IL‐6R, interleukin‐6 receptor; IRAK1, interleukin‐1 receptor‐associated kinase 1; M1, classically activated macrophage; M2, alternatively activated macrophage; miR, microRNA; MSC‐Exos, mesenchymal stem cell‐derived exosomes; MyD88, myeloid differentiation primary response 88; NF‐κB, nuclear factor κB; NLRP3, NOD‐like receptor family pyrin domain‐containing 3; STAT3, signal transducer and activator of transcription 3; TAB2, transforming growth factor β‐activated kinase 1‐binding protein 2; TAK1, transforming growth factor β‐activated kinase 1; Th17, T helper 17 cell; TLR4, Toll‐like receptor 4; TNF‐α, tumor necrosis factor α; TRAF6, tumor necrosis factor receptor‐associated factor 6; Treg, regulatory T cell.

The therapeutic potential of MSC‐Exos in DED has been extensively validated in preclinical research. It exhibits multidimensional interventional capabilities in modulating inflammation, tear film imbalance, and immune regulation during the pathological progression of DED, through the regulation of various signaling pathways, including TLR4/NF‐κB, IRAK1/TRAF6/NF‐κB, STAT3, and the NLRP3 inflammasome, as shown in Figure 3. However, significant gaps remain in the current research on MSC‐Exos‐based DED treatment. First, systematic comparisons of therapeutic efficacy across studies are hampered by two primary factors: (1) source‐dependent heterogeneity: the composition of exosomes depends on their parent cells, and the mesenchymal stem cells used in some experiments originate from different sources, implying potential variations in the active components of each MSC‐Exo, directly impacting functional consistency [88, 89]; (2) technical variability: differences in the cultivation and extraction environments of MSC‐Exos across experiments can lead to variations in their active components and concentrations, directly affecting the reproducibility of their functions [90]. Consequently, the existing findings cannot yet be integrated into a unified inflammatory regulatory network for DED. Second, although exosomes carry diverse bioactive molecules, including RNA and proteins, the therapeutic effects of MSC‐Exos in DED have not yet been resolved to the molecular level. It is difficult to exclude the possibility that other substances within MSC‐Exos may promote inflammatory responses in DED, making it challenging to precisely distinguish the contribution of individual components. Third, given the chronic nature of DED, longitudinal therapeutic dynamics require phase‐specific evaluation. For instance, the STAT3 pathway can inhibit M1 macrophage polarization during the acute phase of DED but may exacerbate corneal fibrosis when overactivated in the chronic phase [91]. Therefore, the inherent heterogeneity of MSC‐Exos combined with the complex pathological progression of DED creates substantial challenges in elucidating their mechanistic actions. This calls for further dissection of their regulatory networks and the development of engineered exosomes capable of stage‐specific responses to optimize therapeutic windows.

### 5.2. Progress in Clinical Research

MSC‐Exos for DED have entered early human testing. At least five interventional trials are registered on ClinicalTrials.gov or national registries, covering bone marrow, adipose, umbilical‐cord, and pluripotent‐stem‐cell sources, mostly via topical eye drops (Table 1) [64, 92–97].

**Table 1:** Registered or published clinical trials of MSC‐ or MSC‐Exos‐based therapy for dry eye disease.

Trial (ref.)	Source/product	Route and regimen	*N*/population	Key outcome measures	Status/key result^a^
NCT04213248 [64]	UC‐MSC‐Exos	Eye drops, QID, 2 week	14 patients/28 eyes; cGVHD‐associated DED	OSDI, TBUT, Schirmer, fluorescein staining, IOP	Completed: significant improvement in TBUT, tear secretion, OSDI, and staining
IRCT20211102052948N1 [92]	UC‐MSC‐Exos	Eye drops, BID, 2 week	8 vs. 8 eyes (PBS control); Sjögren’s‐related DED	OSDI, TBUT, Schirmer, fluorescein, tear EGF/TSP‐1, IL‐6/MMP‐9	Completed (triple‐blind RCT): significant improvement vs. PBS in all measures
NCT06543667 [93]	LSC‐Exos	Eye drops, QID, 3 month	30 (planned); moderate–severe DED	OSDI, tear cytokines, tear secretion, TBUT, redness, BCVA	Recruiting: results not yet available
NCT05738629 [94]	PSC‐MSC‐Exos	Eye drops, QID, 12 week	12; DED postrefractive surgery/blepharospasm	OSDI, TBUT, OSS	Not yet recruiting: results not yet available
NCT04615455 [95]	AD‐MSC	Lacrimal‐gland injection, single dose	54 (20 active/20 placebo/14 observation); Sjögren’s‐associated DED	OSDI (primary), objective signs	Completed RCT: OSDI improved only with active treatment

^a^Trial statuses were verified against the relevant trial registries on July 28, 2026.

Two trials provide the most detailed evidence. A single‐arm phase 1/2 study of UC‐MSC‐Exos eye drops in cGVHD‐associated DED reported significant improvements in tear‐film breakup time, tear secretion, and OSDI after 2 weeks of treatment [64]. A triple‐blind, placebo‐controlled RCT of umbilical‐cord MSC‐Exos in Sjögren’s‐related DED showed parallel clinical improvement along with favorable shifts in tear inflammatory and reparative biomarkers [92]. Two newer trials (LSC‐Exos and PSC‐MSC‐Exos) are extending this approach to other sources and longer treatment durations [93, 94]. For comparison, a randomized trial of whole‐cell adipose‐derived MSCs (not exosomes) improved OSDI only in the active‐treatment arm [95], suggesting that exosomes may offer more consistent objective benefit than whole‐cell therapy, though no trial has directly compared the two.

Across trials, exosome dosing has converged empirically on a similar low range regardless of the source, while treatment duration varies by disease severity and etiology rather than by formal dose‐finding. No dose‐escalation study has been performed, so the dose–response relationship remains an acknowledged evidence gap rather than an established curve.

Source choice (bone marrow/adipose/umbilical cord) also remains empirical (Table 2): BM‐MSC‐Exos are the most extensively characterized and effective via both subconjunctival injection and topical drops [98]; AD‐MSC‐Exos are the easiest to harvest and act substantially through NLRP3 suppression [36, 68]; UC‐MSC‐Exos have the lowest immunogenicity and the only published human DED trials to date [64, 92, 99]. Because nearly all DED studies default to topical delivery rather than systematically comparing routes, no source‐ or route‐specific superiority can yet be claimed.

**Table 2:** Comparison of MSC‐Exos sources relevant to dry eye disease.

Source	Harvest/yield	Immunogenicity	DED‐relevant evidence	Efficacy signal	Key limitation
Bone marrow (BM‐MSC‐Exos)	Invasive aspiration; donor‐dependent yield	Low–moderate	Subconjunctival injection and topical drops; Sjögren’s mouse model [98]	Improved corneal/conjunctival staining, tear volume, goblet cells (both routes)	Invasive harvest limits scalable donation
Adipose tissue (AD‐MSC‐Exos)	Minimally invasive; high yield	Low	Topical eye drops; BAC‐induced mouse DED [36, 68]	NLRP3/cytokine suppression; improved tear volume and TBUT	Source/extraction variability across protocols [88, 89]
Umbilical cord (UC‐MSC‐Exos)	Noninvasive (perinatal tissue); high yield	Lowest	Topical eye drops; only source with published human trials + rat model [64, 92, 99]	Improved TBUT, tear secretion, OSDI, staining	Batch‐to‐batch variability [88, 89]

### 5.3. Delivery System Engineering

With topical ocular administration, fewer than 10% of exosomes penetrate the corneal barrier, while systemic administration results in low target‐tissue distribution due to hepatic and splenic sequestration [100]. Addressing this requires concrete engineering strategies beyond generic “nanotechnology integration.” Surface modification with cationic or cell‐penetrating peptides (e.g., TAT and arginine‐rich motifs) electrostatically loosens corneal tight junctions and has markedly enhanced trans‐corneal exosome penetration compared with unmodified vesicles [101]. For carrier combination strategies, thermosensitive chitosan or GelMA hydrogels loaded with MSC‐Exos prolong ocular‐surface retention and sustain release over days rather than minutes, improving repair outcomes versus exosome eye drops alone in corneal‐injury models [102]; liposome–exosome hybrid nanovesicles combine the loading capacity of liposomes with the natural targeting of exosome membrane proteins to improve both retention and tissue‐specific uptake [103]; microneedle‐loaded exosome systems, so far demonstrated for glaucoma rather than DED, physically bypass rather than enhance penetration of the corneoscleral barrier, trading minimal invasiveness for a depot effect [104]. Hydrogels and surface peptide modification have the most direct anterior‐segment relevance for DED, whereas microneedles are better suited to scleral/periocular depot delivery; head‐to‐head in vivo comparisons of targeting efficiency and safety across these strategies specifically in DED models are still lacking and should be flagged as a gap rather than treated as interchangeable. Most such multifunctional nanoparticle‐based delivery platforms remain in preclinical stages, necessitating large‐scale animal studies and safety/efficacy validation before clinical adoption; furthermore, the binding patterns between MSC‐Exos and ocular surface epithelial cells, their cellular uptake mechanisms, and intracellular clearance time remain poorly understood, leading to unpredictable effective drug half‐lives and uncertain duration of action [105].

### 5.4. DED Subtype Specificity and Long‐Term Safety

Subtype‐specific efficacy data remain limited, but the first head‐to‐head comparison is informative: a first‐in‐human pilot trial of UC‐MSC eye drops in both non‐SS (*n* = 11) and SS (*n* = 5) dry eye found clinical and tear‐film improvements were more pronounced in non‐Sjögren’s patients, with Sjögren’s patients showing a measurable but comparatively smaller benefit [106], suggesting the systemic autoimmune component of secondary/Sjögren’s‐associated DED may partially blunt the local anti‐inflammatory effect of MSC‐Exos relative to nonautoimmune aqueous‐deficient DED. No published study has yet isolated evaporative (MGD‐driven) DED as a distinct treatment arm, so efficacy claims for that subtype remain extrapolated rather than directly demonstrated.

Regarding long‐term safety, MSC‐Exos avoid the risks of ectopic tissue formation and direct malignant transformation associated with whole‐cell MSC transplantation. However, published DED trials have reported follow‐up periods of only 2 weeks to 12 months, and no multiyear human safety data are currently available. Moreover, exosomes derived from certain MSC sources have been reported to promote tumor growth in nonocular experimental settings [107]. Importantly, these findings were obtained in tumor‐bearing nonocular models and may not directly translate to topical ocular administration, which would generally be expected to produce substantially lower systemic exposure than systemic delivery. Nevertheless, reduced systemic exposure does not eliminate potential risk; future DED trials should therefore assess ocular biodistribution, systemic exposure, and long‐term tumor‐related outcomes. Accordingly, the assumption of low tumorigenicity should not yet be considered established.

In summary, MSC‐Exos have demonstrated significant therapeutic potential for DED, but their clinical advancement faces numerous technical and scientific challenges. Future research should prioritize standardization of exosome preparation, deeper mechanistic characterization, and rigorous safety assessment in clinical settings. With continued advances in nanomedicine, gene editing, and bioengineering, the clinical translation prospects of MSC‐Exos as a novel biological therapy are highly promising.

## 6. Conclusion

MSC‐Exos represent a transformative therapeutic paradigm for DED, orchestrating synergistic suppression of ocular surface inflammation through multipathway modulation—including TLR4/NF‐κB, IRAK1/TRAF6, STAT3, and the NLRP3 inflammasome—while unveiling novel mechanisms of gut–eye axis‐driven Th17/Treg balance and FBXW7‐ubiquitination degradation. Despite compelling preclinical evidence, clinical translation faces critical barriers: exosomal heterogeneity impedes standardization, suboptimal corneal penetration limits delivery efficiency, and long‐term safety and efficacy have yet to be established. To surmount these challenges, future development must integrate nanotechnology for enhanced tissue targeting, CRISPR‐based engineering to standardize anti‐inflammatory cargo (e.g., miR‐146a‐5p and miR‐223‐3p), and gut microbiome modulation to amplify immunoregulatory effects. Integrating these bioengineering strategies will advance MSC‐Exos from mechanistic promise to clinically viable precision therapeutics, ultimately transforming DED management.

## Author Contributions

Conceptualization: Jiahuan Yi, Shigang Xia, Jia Zhang, and Qiguo Xiao. Data curation and investigation: Jiahuan Yi, Fangfang Lu, Yale Tian, Yu Wang, Zihe Zhang, Xujie Deng, and Zhi Wang. Funding acquisition: Fangfang Lu and Jia Zhang. Visualization: Jiahuan Yi and Fangfang Lu. Supervision and project administration: Shigang Xia, Jia Zhang, and Qiguo Xiao. Writing – original draft: Jiahuan Yi, Fangfang Lu, Yale Tian, Yu Wang, Zihe Zhang, Xujie Deng, and Zhi Wang. Writing – review and editing: Shigang Xia, Jia Zhang, and Qiguo Xiao.

## Funding

This work was supported by the Hunan Provincial Natural Science Foundation (Grant 2025JJ81029) and the Hunan Provincial Natural Science Foundation (Grant 2025JJ81006).

## Disclosure

The funders had no role in study design, data collection and interpretation, or the decision to submit the work for publication. All contributing authors have been listed in this article. No third‐party service agencies were involved in the writing of the manuscript.

## Consent

The authors have nothing to report.

## Conflicts of Interest

The authors declare no conflicts of interest.

## Supporting Information

Additional supporting information can be found online in the Supporting Information section.

## Supporting information


**Supporting Information** Appendix S1: Search strategy and study selection criteria.

## Data Availability

Data sharing is not applicable to this article, as no datasets were generated or analyzed during the current study.
