# Supplementary material for: Mechanisms of Mesenchymal Stem Cell‐Derived Exosomes in Dry Eye Disease: From Inflammation Pathways to Therapeutic Prospects
Source: Stem Cells Int. 2026 Sep 9;2026:2026673. doi: 10.1155/sci/2026673 (PMC13555284; doi:10.1155/sci/2026673)
Supplement: Supplementary file 1 [file SCI-2026-2026673-s001.docx]

**Supplementary Appendix S1. Search Strategy and Study Selection Criteria**

PubMed and Embase were searched from January 2010 to June 2026, supplemented by hand-searching of reference lists and trial registries (ClinicalTrials.gov and national registries) for clinical trials. Search terms combined ("mesenchymal stem cell" OR "mesenchymal stromal cell") AND ("exosome" OR "extracellular vesicle") AND ("dry eye" OR "keratoconjunctivitis sicca" OR "Sjögren"), supplemented with pathway-specific terms (TLR4, NF-κB, IRAK1/TRAF6, STAT3, NLRP3, FBXW7, gut-eye axis, Th17/Treg, microRNA) to ensure mechanistic coverage. Database-specific syntax (MeSH/Emtree, truncation, field tags) was adjusted accordingly, and results were de-duplicated before screening.

**Inclusion:** original research or peer-reviewed reviews on MSC-Exos (any source) in dry eye disease, Sjögren's-associated dry eye, or closely related ocular surface inflammatory conditions; studies addressing the specific molecular pathways discussed in this review; registered or published clinical trials of MSC- or MSC-Exos-based DED therapy; English-language, peer-reviewed publications.

**Exclusion:** whole-cell MSC studies without an exosome/EV component (unless used as a direct comparator); conference abstracts, preprints, case reports lacking quantitative data, and non-English publications; exosomes from non-MSC sources unless used as an experimental comparator; duplicate reports of the same dataset.

Titles/abstracts were screened for relevance followed by full-text review of eligible records. As this is a narrative rather than systematic review, study selection prioritized mechanistic completeness over exhaustive literature capture; this scope limitation is noted for transparency.
